# Comparison of DASH diet score and total antioxidant capacity of diet on serum levels of TMPRSS‐2, inflammatory biomarkers, and disease severity in COVID‐19 patients: A case–control study

**DOI:** 10.1002/fsn3.4024

**Published:** 2024-02-14

**Authors:** Fatemeh Dibaseresht, Mohammad Alizadeh, Jalal Moludi

**Affiliations:** ^1^ Department of Nutrition, Faculty of Nutrition and Food Science Tabriz University of Medical Science Tabriz Iran; ^2^ Department of Clinical Nutrition, Nutrition Research Center, Faculty of Nutrition and Food Science Tabriz University of Medical Sciences Tabriz Iran; ^3^ Department of Nutritional Sciences, School of Nutritional Sciences and Food Technology Kermanshah University of Medical Sciences Kermanshah Iran

**Keywords:** DASH diet score, inflammatory biomarkers, severity of COVID‐19, TAC, TMPRSS‐2

## Abstract

There is evidence that healthy diets improve the immune system and lessen the severity of infectious diseases such as COVID‐19. We have investigated whether the dietary total antioxidant capacity (TAC) and dietary approach to stop hypertension (DASH) score could be associated with the occurrence and clinical outcomes of COVID‐19. This case–control study included 120 adults who were admitted to the hospital. Dietary TAC and DASH diet scores were determined by a 138‐item semi‐quantitative food frequency questionnaire (FFQ). Inflammation‐related markers including C‐reactive protein (CRP) and transmembrane protease serine 2 (TMPRSS‐2) differential were measured. Also, using chest radiology criteria, the severity of the disease was evaluated. The mean CRP values in the lowest and highest tertiles of either dietary TAC or DASH diet scores were 9.44 ± 11.26 and 3.52 ± 4.83 mg/dL (*p* = .003) or 9.04 ± 11.23 and 4.40 ± 6.23 mg/dL (*p* = .013), respectively. Individuals with higher dietary TAC were at a lower risk of COVID‐19 (OR: 0.06, *p* < ·0001). Individuals with greater DASH diet scores were also at decreased odds of COVID‐19 (OR: 0.12, *p* < ·0001). No significant associations were found between dietary TAC and DASH diet scores with severity of COVID‐19 disease, CRP, or TMPRSS‐2 (*p* > 0.05). The study found that adherence to a diet with higher dietary TAC and DASH diet scores may be protective against COVID‐19 and improve outcomes of the disease. More research is needed to corroborate these findings.

## Graphical abstract

We have investigated whether the dietary total antioxidant capacity (TAC) and dietary approach to stop hypertension (DASH) score could be associated with occurrence and clinical outcomes of COVID‐19. The study found that adherence to a diet with higher dietary TAC and DASH diet scores may be protective against COVID‐19 and improve outcomes of the disease. More research is needed to corroborate these findings.

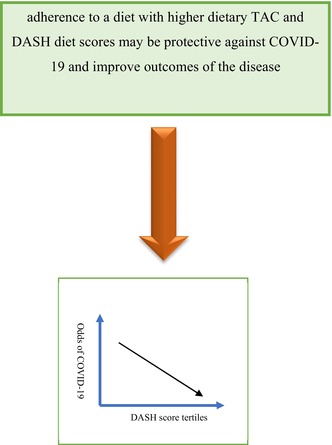

## 1 INTRODUCTION

Despite substantial advancements in medical science, world health and well‐being are threatened by periodic infectious diseases, particularly viral epidemics such as influenza, pneumonia, norovirus, and severe acute respiratory syndrome (Cassell & Mekalanos, 2001; Kanauchi et al., 2018). The latest outbreak of these infections was the 2019 Coronavirus (COVID‐19) pandemic, which imposed significant impacts on lifestyles, incidence of metabolic diseases, and global health status (Brooks et al., 2020).

Nutritional status is a contributing factor for prevention and protection of various infectious diseases including COVID‐19 (Laviano et al., 2020). Based on evidence, it has been widely emphasized that adequate nutritional status and dietary habits are important for preventing the occurrence of COVID‐19 and other viral infections, through modulating the inflammatory status of the patient (Fernández‐Quintela et al., 2020).

Diet and nutrition influence immunologic performance and the severity and incidence of infection. Diet, nutritional status, infection, and immune function all have reciprocal interactions so that alteration in one component affects the others. Variety of macro‐, micro‐, and phytonutrients are essential for a balanced immunologic function, and nutritional deficiencies are associated with increased host vulnerability to many infections including viral pathogens and increased clinical course of the diseases (Chandra, 1996). With regard to this fact, vitamins including A, B6, B12, E, C, and D, as well as folate and trace minerals such as zinc, copper, iron, magnesium, and selenium are necessary to promote both the innate and adaptive immune responses. On the other hand, a shortage of micronutrients impairs the immune system and decreases resistance to many pathogens (Carr & Maggini, 2017; Glaab & Ostaszewski, 2020). Thus, dietary intake can be substantially effective in the context of severe COVID‐19 as an uncontrolled inflammatory status (Calder et al., 2020).

Transmembrane Protease Serine‐2 (TMPRSS‐2), an enzyme located in epithelial cells of the lung, is suggested to facilitate attachment of coronavirus to the host cell's receptors through a spike protein (Letko et al., 2020). This also increases viral replication, as well as the production of inflammatory cytokines and chemokines (Beeraka et al., 2020), which leads to an inflammatory cytokine storm, defects in the immune system, and ultimately devastating consequences such as multi‐organ dysfunction (Huang et al., 2020; Tay et al., 2020; Zhou et al., 2020).

Several modulators, including nutrition, have an impact on an inflammatory state (Calder et al., 2013). The total antioxidant capacity (TAC) has been introduced as an index to assess the cumulative effects of antioxidants from food in scavenging free radicals, because there are several antioxidant compounds in foods and the capacity of a specific food is due to their possible interaction (Puchau et al., 2009; Wu et al., 2004). The elevated level of oxidative stress and decreased dietary TAC can exacerbate the disease severity in hospitalized COVID‐19 patients (Karkhanei et al., 2021; Yaghoubi et al., 2022).

Among patients with or at risk of infectious diseases, adherence to specific dietary patterns is very important due to potential interactions between dietary foods/nutrients and their final impact on host defense system (Soltani et al., 2020). Dietary approaches to stop hypertension (DASH) diet is known as a diet that recommends consuming fruits, vegetables, and low‐fat dairy products and restricting the consumption of meats, sugar‐sweetened beverages, and saturated fat (Appel et al., 1997). Recent research has revealed that the DASH diet significantly diminishes indicators of inflammation such as C‐reactive protein (CRP) levels (Niknam et al., 2015; Saneei, Hashemipour, et al., 2014). Thus, it is thought that the diet may be a more effective predictor of disease risk (Moludi et al., 2020) (Soltani et al., 2020).

Even though there is no clinically proven treatment for COVID‐19, it appears that optimum intake of nutrients by a healthy balanced diet may be an efficient strategy for managing the ongoing risk of infectious diseases like COVID‐19. Thus, this case–control investigation was conducted to examine the association of dietary TAC and DASH diet scores with inflammatory biomarkers, incidence, and disease severity of COVID‐19.

## 2 MATERIALS AND METHODS

### 2.1 Study design

**FIGURE 1:**
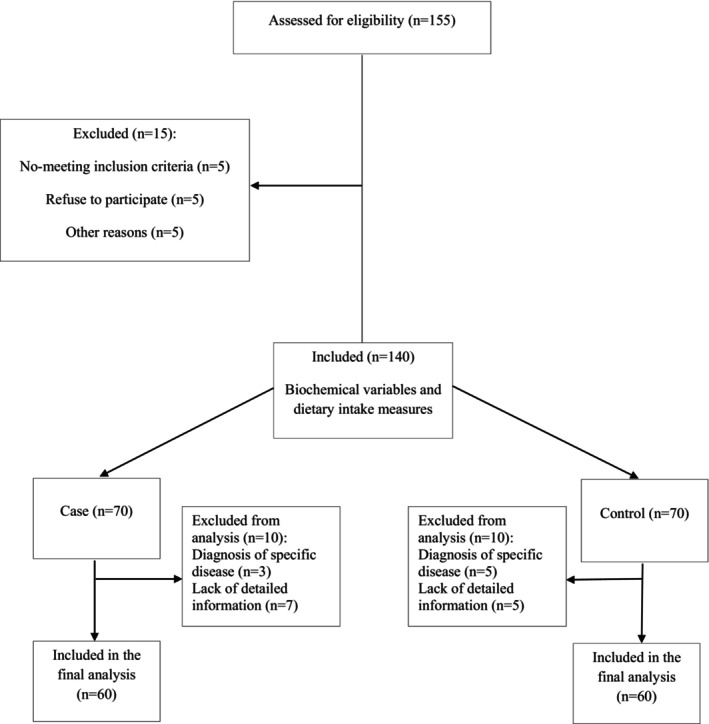
Flowchart.

This article was a case–control investigation carried out in June and July 2020. 120 individuals (60 COVID‐19 patients and 60 healthy controls) were included in the study analysis (Figure 1). Following recovery, patients (male and female) were invited to take part in the study if they so desired. We requested relatives of patients to help subjects who could not answer the questionnaire fairly. The age group of 30–70 years was considered as the inclusion criteria. The case group was chosen from patients without being unconscious who had tested positive for COVID‐19 and had been admitted to Imam Reza Hospital, in Tabriz City, Azerbaijan province, and Golestan Hospital, in Kermanshah City, Kermanshah province, both in Iran. The COVID‐19 infection of patients was confirmed by clinical symptoms, RT‐PCR (reverse transcription polymerase chain reaction) test, and chest tomography, in accordance with WHO provisional guidelines (Lechien et al., 2020). Based on clinical assessments, laboratory findings, and chest radiography results, all patients suffering from COVID‐19 were classified as mild, moderate, and severe cases. The control group consisted of asymptomatic patients who tested negative for COVID‐19 and received referrals to the outpatient ward for multiple kinds of medical conditions (trauma, injuries, fractures, sprains, and skin illnesses) between June and July 2020.

The exclusion criteria were history of inflammatory illnesses, for instance, multiple sclerosis, rheumatoid arthritis, cancer, endocrine, liver and renal disorders, cardiovascular diseases, diabetes, pregnancy, lactation, utilizing immunosuppressive medications, history of weight loss in the last month, incompletely answering the questionnaire, dietary under, and over‐reporting (energy intake more than 800 or less than 4200 kcal per day), following certain special diets such as weight loss or a vegetarian diet, and taking vitamin, antioxidant, or probiotic supplements in the previous 6 months.

The Ethics Board of Tabriz University of Medical Sciences (IR.TBZMED.REC.1401.446) accepted the research protocol. The informed consent form was filled by all participants.

### 2.2 General and anthropometric measurements

Demographic data, including gender, age, educational levels, physical activity, smoking status, dietary habits, medical history, and nutritional supplement history, were extracted through self‐constructed questionnaire. Weight and height measurements upon admission were either the patient's self‐reporting or conducted by a trained nutritionist. The weight and height of participants were estimated using a digital scale in light clothing with an accuracy of 0.1 kg (SECA, Hamburg, Germany), and a tape measure in standing without shoes to the nearest 0.5 cm (SECA, Hamburg, Germany), respectively. The usual dietary intake of the participants was collected using a 138‐item semi‐quantitative FFQ (Food Frequency Questionnaire) via face‐to‐face interviews with expert nutritionists. The mentioned FFQ that was designed and validated for the Iranian population comprises several items with standard portion sizes, which are typically consumed by Iranians (Mirmiran et al., 2010). For each food item, participants were requested to state their frequency of consumption during the last year (daily, weekly, monthly, or yearly). Subsequently using household measurements, the portion size of consumed food was converted into grams per day for 1000 Kcal, and dietary intake was analyzed by Nutritionist IV software (Nutritional Database Manager 4·0·1, First Data Bank).

### 2.3 Biochemical assessments

Erythrocyte sedimentation rate (ESR) and C‐reactive protein (CRP) measurements were conducted by Westergren and immunoturbidimetric technique (Pars Azmoon), respectively. The complete blood count test was examined using an autoanalyzer (sysmes XS‐500i). The serum level of TMPRSS‐2 was also measured using special human kits (Zell BIO, Germany) via the ELISA method.

### 2.4 Assessment of dietary TAC

The ferric‐reducing ability of plasma (FRAP) method was used to calculate dietary TAC values (Benzie & Strain, 1996). FRAP is based on the antioxidant power of reducing Fe^+3^ (ferric) to Fe^+2^ (ferrous). To calculate dietary total antioxidant capacity for each participant, the ferric‐reducing antioxidant potency assay of all dietary components formerly published was added together (Halvorsen et al., 2002, 2006; Pellegrini et al., 2003, 2006). Dietary TAC has been calculated in millimoles per 100 grams of food. To give a TAC value to items that were absent in earlier reports, data from a similar food item were utilized as a reference. When cooked food TAC values were not available, fresh food TAC values were utilized to determine the dietary TAC (Puchau et al., 2009).

### 2.5 Assessment of DASH diet score

The DASH diet score was computed using method set by (Fung et al., 2008). This method consists of eight components and focuses on a higher intake of fruits, vegetables, nuts, legumes, low‐fat dairy products, and whole grains, and a lower intake of sodium, sweetened beverages, and red and processed meats. The classification of the study subjects was based on the quintile of each mentioned diet item. Each quintile was assigned a score ranging from 1 to 5. The quintile ranking was based on the following: for fruits, vegetables, nuts and legumes, low‐fat dairy products, and whole grains highest quintile was given 5 points, while the lowest quintile was given 1 point. For sodium, red, and processed meats, and sweetened beverages, the scoring was reversed. Finally, DASH diet score (8–40) was calculated by adding the eight food component scores (Fung et al., 2008; Nilsson et al., 2019). A higher score indicates higher adherence to the DASH diet and vice versa.

### 2.6 Statistical analysis

Statistical data analyses were run by IBM SPSS statistics 21 (SPSS Inc., Chicago). The *p*‐value <0.05 was considered as the statistical significance threshold. The variables were evaluated in terms of normal and non‐normal distribution through the Kolmogorov–Smirnov statistical test. The variables with normal and non‐normal distributions were presented as mean and standard deviation (SD), and median and interquartile range (IQR), respectively. Categorical variables were reported as frequency and percentage. The comparison of case and control groups was conducted by Chi‐square tests for qualitative variables, independent sample t‐tests for quantitative variables with normal distribution, and Mann–Whitney U tests for quantitative variables with non‐normal distribution. The comparison of subjects among the tertiles of DASH diet score and dietary TAC was carried out using one‐way analysis of variance (ANOVA) for continuous variables. To control the impact of possible confounding factors, two adjusted models (model 1: adjusted for sex, age, BMI, energy intake; model 2: adjusted for model 1 plus diabetes and hypertension) were applied in addition to a crude model (unadjusted). The relationship between dietary TAC and DASH diet scores with odds of COVID‐19 disease was examined as OR and 95% CI based on binary logistic regression. Investigating the relationship between dietary TAC and DASH diet scores with the severity of disease in COVID‐19 patients was performed using multivariate logistic regression. Linear regression was also applied to explore whether there was a link between dietary TAC and DASH diet scores with inflammation‐related biomarkers in patients with COVID‐19 infection.

## 3 RESULTS

### 3.1 Characteristics of the participants

Table 1 provides the basic characteristics of the study subjects by the case and control groups. The median age for the case group was 64 (interquartile range: 55, 69), while for the control group, it was 58 (interquartile range: 47, 65) (*p* = .004). No significant statistical difference was found between the case and control groups in terms of weight, height, body mass index (BMI), sex, and medical history. Non‐smokers were more dominant in both groups, with 70.5% of the controls and 71.7% of the cases being non‐smokers. Similarly, non‐diabetics were more prevalent than individuals with diabetes, accounting for 85.2% of the controls and 78.3% of the cases. The most common comorbidity in both groups was hypertension, with 65.6% of the controls and 56.7% of the cases having this condition.

**TABLE 1:** Characteristics of study subjects.

Variable		Healthy	COVID‐19	*p*
Age (year)^a^		58 (47, 65)	64 (55, 69)	.004
Weight (kg)^a^		70 (66, 82)	73 (65, 88)	.202
Height (cm)^a^		166 (156, 172)	167.75 (162, 173.75)	.060
BMI (kg/m^2^)^a^		26.08 (23.70, 28.35)	26.90 (24.56, 29.61)	.544
CRP (mg/dl)^a^		2.5 (1.9, 3)	9 (3, 31)	<.0001
TMPRSS‐2^a^		145.95 (139.42, 151.22)	144.55 (129.90, 150.82)	.171
TAC^b^		657.36 ± 194.64	507.93 ± 87.65	<.0001
DASH diet score^b^		26.39 ± 3.99	23.46 ± 3.18	<.0001
Sex^c^	Male	43 (70.5%)	42 (70%)	.953
	Female	18 (29.5%)	18 (30%)	
Activity^c^	Low	38 (62.3%)	7 (11.7%)	<.0001
	Moderate	20 (32.8%)	49 (81.7%)	
	High	3 (4.9%)	4 (6.7%)	
Smoking^c^	Yes	18 (29.5%)	17 (28.3%)	.887
	No	43 (70.5%)	43 (71.7%)	
Diabetes^c^	Yes	9 (14.8.5%)	13 (21.7%)	.324
	No	52 (85.2%)	47 (78.3%)	
Hypertension^c^	Yes	40 (65.6%)	34 (56.7%)	.315
	No	21 (34.4%)	26 (43.3%)	

Abbreviations: BMI, Body mass index; CRP, C‐reactive protein; DASH score, Dietary approach to stop hypertension score; TAC, Total antioxidant capacity; TMPRSS‐2, Transmembrane protease serin‐2.

^a^ Values are expressed as median (interquartile range) and *p‐value* based on Mann–Whitney *U* test.

^b^ Values are expressed as Mean ± SD and *p‐valu*e based on independent sample *t*‐test.

^c^ Values are expressed as frequency (%) and *p‐value* based on chi‐squared test.

In addition, Table 1 shows findings on dietary TAC, DASH diet score, and inflammatory markers, including CRP and TMPRSS‐2. The mean CRP was significantly higher in COVID‐19 patients (*p* < ·001), but there was no significant difference in terms of TMPRSS‐2 between the two groups (*p* = .171). The dietary TAC varied from 385 (fewer antioxidants) to 1159 (more antioxidants), and the DASH diet score varied from 16 (less adherence to the DASH diet) to 33 (more adherence to the DASH diet). The mean ± standard deviation (SD) of dietary TAC for the case and control group were 657.36 ± 194.64 and 507.93 ± 87.65, respectively (*p* < .0001). The mean ± standard deviation (SD) of the DASH diet score for the case and control groups were 26.39 ± 3.99 and 23.46 ± 3.18, respectively (*p* < .0001).

Table 2 compared inflammatory and clinical markers across the tertiles of dietary TAC and DASH diet scores. The mean CRP was significantly lower in the highest tertile of dietary TAC than the lowest tertile (tertile 1: 9.44 ± 11.26; tertile 2: 10.90 ± 11.72; tertile 3: 3.52 ± 4.83; *p* = .003). Patients in the highest tertile of dietary TAC had shorter hospital time than those in the lowest tertile (tertile 1: 8.97 ± 3.26; tertile 2: 8.53 ± 3.97; tertile 3: 6.87 ± 3.64; *p* = .037). Likewise, the mean CRP was significantly lower in the highest tertile of the DASH diet score than the lowest tertile (tertile 1: 9.04 ± 11.23; tertile 2: 10.83 ± 11.78; tertile 3: 4.40 ± 6.23; *p* = .013). Patients in the highest tertile of DASH diet score had shorter hospital time than those in the lowest tertile (tertile 1: 9.42 ± 3.13; tertile 2: 8.15 ± 3.94; tertile 3: 7.14 ± 3.70; *p* = .040). ESR, WBC, TMPRSS‐2, and ICU time did not indicate a statistical difference across the tertiles of dietary TAC and DASH diet scores (*p* > .05).

**TABLE 2:** Comparison of inflammatory and clinical markers in tertiles of dietary TAC and DASH diet scores.

Variables	TAC tertile 1 (<479.79)	TAC tertile 2 (479.79–617.21)	TAC tertile 3 (>617.21)	*p*‐trend
CRP (mg/dl)	9.44 ± 11.26	10.90 ± 11.72	3.52 ± 4.83	.003
ESR (mm/h)	20.54 ± 22.48	23.23 ± 21.19	23.60 ± 28.66	.910
WBC count	7861.46 ± 4499.26	7987.70 ± 3072.07	6925 ± 2126.61	.880
TMPRSS‐2	146.78 ± 6.72	141.01 ± 12.45	147.31 ± 32.96	.564
Hospital time (d)	8.97 ± 3.26	8.53 ± 3.97	6.87 ± 3.64	.037
ICU time (d)	2.08 ± 3.2	2.18 ± 3.06	3 ± 4.24	.839

Variables	DASH score tertile 1 (<23)	DASH score tertile 2 (23–27)	DASH score tertile 3 (>27)	*p*‐trend
CRP (mg/dl)	9.04 ± 11.23	10.83 ± 11.78	4.40 ± 6.23	.013
ESR (mm/h)	21.05 ± 23.71	24.42 ± 21.34	18.33 ± 22.17	.772
WBC count	7683.27 ± 4301.48	8213.80 ± 3861/25	7318 ± 1905.41	.831
TMPRSS‐2	147.25 ± 7.47	140.86 ± 12.66	147.02 ± 31.47	.574
Hospital time (d)	9.42 ± 3.13	8.15 ± 3.94	7.14 ± 3.70	.040
ICU time (d)	2 ± 3.01	2.33 ± 3.15	2.37 ± 3.54	.932

*Note*: Values are expressed as Mean ± SD and *p‐valu*e based on one‐way analysis of variance (ANOVA).

Abbreviations: CRP, C‐reactive protein; DASH, Dietary approach to stop hypertension; ESR, erythrocyte sedimentation rate; TAC, Total antioxidant capacity; TMPRSS‐2, Transmembrane protease serin‐2; WBC, white blood cell.

### 3.2 Association of dietary TAC and DASH diet scores with odds of COVID‐19 and disease severity

Table 3 presented the COVID‐19 OR and 95% CI for each tertile of dietary TAC and DASH diet scores. The odds of COVID‐19 in the third tertile of dietary TAC were 0.06 (*p* < .0001) in the first tertile. After adjustment for all confounders, the odds of COVID‐19 in tertile 3 were 0.05 of tertile 1 (*p* < ·0001). Similarly, the odds of COVID‐19 in the third tertile of the DASH diet score were 0.12 (*p* < .0001) in the first tertile. After adjustments for all cofounders the odds of COVID‐19 in tertile 3 were 0.09 of tertile 1 (*p* < .0001).

**TABLE 3:** Association between dietary TAC and DASH diet scores with odds of COVID‐19 disease.

		TAC tertile 1 (<479.79)	TAC tertile 2 (479.79–617.21)	TAC tertile 3 (>617.21)	*p*‐trend
		Odds ratio (95% CI)	Odds ratio (95% CI)	Odds ratio (95% CI)	
COVID‐19	Crude	1.00	0.59 (0.23, 1.51)	0.06 (0.02, 0.20)	<.0001
	Model 1	1.00	0.64 (0.23, 1.74)	0.04 (0.01, 0.17)	<.0001
	Model 2	1.00	0.63 (0.22, 1.77)	0.05 (0.01, 0.17)	<.0001

		DASH score tertile 1 (<23)	DASH score tertile 2 (23–27)	DASH score tertile 3 (>27)	*p*‐trend
		Odds ratio (95% CI)	Odds ratio (95% CI)	Odds ratio (95% CI)	
COVID‐19	Crude	1.00	0.63 (0.24, 1.66)	0.12 (0.04, 0.34)	<.0001
	Model 1	1.00	0.56 (0.20, 1.55)	0.09 (0.03, 0.29)	<.0001
	Model 2	1.00	0.56 (0.20, 1.59)	0.09 (0.03, 0.30)	<.0001

*Note*: Values are expressed as OR and 95% CI based on binary logistic regression.

Model 1: adjusted for sex, age, BMI, energy intake.

Model 2: adjusted for model 1 plus diabetes and hypertension.

Abbreviations: DASH, Dietary approach to stop hypertension; TAC, Total antioxidant capacity.

As indicated in Table 4, there was no significant association between dietary TAC and DASH diet scores with the severity of COVID‐19 disease (OR = 1.002, *p* = .604) (OR = 1.094, *p* = .449), respectively. Again, adjustment for all confound variables including sex, age, BMI, energy intake, diabetes, and hypertension did not show any significant difference (OR = 1.004, *p* = .470) (OR = 1.152, *p* = .37).

**TABLE 4:** Association of dietary TAC and DASH diet scores with severity of disease in COVID‐19 patients.

Variables		Mild		Moderate		Critical	
		Odds ratio (95% CI)	*p*	Odds ratio (95% CI)	*p*	Odds ratio (95% CI)	*p*
TAC	Crude	1.00		0.997 (0.991, 1.004)	.436	1.002 (0.994, 1.010)	.604
	Model 1	1.00		0.998 (0.990, 1.005)	.524	1.002 (0.993, 1.011)	.631
	Model 2	1.00		0.998 (0.990, 1.005)	.542	1.004 (0.994, 1.014)	.470
DASH diet score	Crude	1.00		0.960 (0.800, 1.152)	.661	1.094 (0.867, 1.381)	.449
	Model 1	1.00		0.973 (0.799, 1.185)	.787	1.101 (0.848, 1.430)	.469
	Model 2	1.00		0.982 (0.790, 1.207)	.864	1.152 (0.866, 1.532)	.333

*Note*: Values are expressed as OR and 95% CI based on multinomial logistic regression.

Model 1: adjusted for sex, age, BMI, energy intake.

Model 2: adjusted for model 1 plus diabetes and hypertension.

Abbreviations: DASH, Dietary approach to stop hypertension; TAC, Total antioxidant capacity.

### 3.3 Association of dietary TAC and DASH diet scores with inflammatory and clinical parameters in COVID‐19 patients

Because the controls were not admitted to the intensive care unit, the data in Table 5 were restricted to COVID‐19 patients. Dietary TAC was not significantly associated with hospital time (*β* = −.010, *p* = .160), ICU time (*β* = .009, *p* = .151), CRP (*β* = .026, *p* = .362), and TMPRSS‐2 (*β* = −.035, *p* = .376). Similar to the same results, there was no significant association between the DASH diet score with hospital time (*β* = −.213, *p* = .286), ICU time (*β* = .212, *p* = .212), CRP (*β* = .664, *p* = .402), and TMPRSS‐2 (*β* = −1.403, *p* = .280).

**TABLE 5:** Association of dietary TAC and DASH diet scores with inflammatory and clinical markers in COVID‐19 patients (*n* = 60)*

Variables		TAC		DASH diet score	
		*β* (95% CI)	*p*	*β* (95% CI)	*p*
CRP (mg/dl)	Crude	0.026 (−0.031, 0.083)	.362	0.664 (−0.910, 2.239)	.402
	Model 1	0.032 (−0.025, 0.088)	.268	0.830 (−0.747, 2.407)	.296
	Model 2	0.036 (−0.023, 0.094)	.223	0.926 (−0.692, 2.544)	.256
ESR (mm/h)	Crude	0.034 (−0.040, 0.109)	.360	0.767 (−1.291, 2.826)	.459
	Model 1	0.044 (−0.030, 0.118)	.236	1.094 (−0.960, 3.148)	.290
	Model 2	0.042 (−0.034, 0.117)	.277	0.983 (−1.121, 3.088)	.353
WBC count	Crude	5.090 (−8.874, 19.054)	.469	129.095 (−255.980, 514.170)	.505
	Model 1	5.509 (−8.726, 19.745)	.441	144.786 (−250.354, 539.925)	.466
	Model 2	2.564 (−11.310, 16,437)	.712	69.987 (−314.241, 454.214)	.716
TMPRSS‐2	Crude	−0.035 (−0.113, 0.044)	.376	−1.403 (−3.993, 1.187)	.280
	Model 1	−0.039 (−0.119, 0.041)	.331	−1.475 (−4.106, 1.156)	.264
	Model 2	−0.037 (−0.115, 0.040)	.334	−1.511 (−4.053, 1.031)	.236
Hospital time (d)	Crude	−0.010 (−0.024, 0.004)	.160	−0.213 (−0.069, 0.183)	.286
	Model 1	−0.010 (−0.023, 0.004)	.163	−0.198 (−0.581, 0.185)	.305
	Model 2	−0.012 (−0.025, 0.001)	.076	−0.268 (−0.638, 0.103)	.153
ICU time (d)	Crude	0.009 (−0.003, 0.012)	.151	0.212 (−0.124, 0.549)	.212
	Model 1	0.011 (−0.001, 0.022)	.064	0.281 (−0.037, 0.599)	.082
	Model 2	0.011 (−0.001, 0.022)	.080	0.272 (−0.056, 0.600)	.102

*Note*: Values are expressed as *β* and 95% CI based on linear regression.

Model 1: adjusted for sex, age, BMI, energy intake.

Model 2: adjusted for model 1 plus diabetes and hypertension.

Abbreviations: CRP, C‐reactive protein; DASH, Dietary approach to stop hypertension; ESR, erythrocyte sedimentation rate; TAC, Total antioxidant capacity; TMPRSS‐2, Transmembrane protease serin‐2; WBC, white blood cell.

* Analysis limited to case group.

## 4 DISCUSSION

One of the purposes of this article was to examine the relationship between TAC and DASH diet scores, and their potential impact on the risk of contracting COVID‐19. The results obtained from this investigation demonstrated that individuals in the higher tertiles of dietary TAC and DASH diet scores had a lower risk of developing COVID‐19 compared to those in the lower tertiles. This suggests that a diet rich in antioxidants and adhering to the DASH diet may offer some protection against COVID‐19. However, the study did not find a significant link between dietary TAC and DASH diet scores with the severity of COVID‐19. There were no significant differences in hospital time, ICU time, CRP, and TMPRSS levels based on these dietary factors. This implies that while a higher dietary TAC and DASH diet scores may reduce the risk of contracting COVID‐19, they may not necessarily lead to significant improvements in inflammatory biomarkers or a shorter duration of hospital stay in COVID‐19 patients.

Several studies have indicated that consuming legumes rich in fiber and adhering to the DASH diet can significantly reduce inflammation, as evidenced by lower serum levels of hs‐CRP (Salehi‐Abargouei et al., 2015; Soltani et al., 2018). One possible explanation for these findings is that a high‐fiber, low‐glycemic index diet can decrease inflammation and the risk of COVID‐19 by delaying glucose absorption, modulating gut flora, improving intestinal mucosal integrity, reducing gut permeability, and inhibiting the production of inflammatory mediators (Estruch et al., 2009; Parada Venegas et al., 2019; Tan et al., 2014). The study findings showed that there was no significant association between dietary DASH and TAC index and the duration of hospital stay, duration of intensive care unit (ICU) stay, levels of CRP, and TMPRSS‐2. The findings regarding the lack of association between dietary TAC and clinical outcomes in COVID‐19 patients are noteworthy. Previous research has proposed that antioxidants may be involved in mitigating the inflammatory response and have an effect on reducing the severity of viral infections (De Flora et al., 2021). However, in this study, dietary TAC did not show a significant impact on hospital time, ICU time, CRP levels, or TMPRSS‐2 expression in COVID‐19 patients. It should be noted that dietary TAC represents the overall antioxidant capacity of the diet, including various antioxidants derived from food sources (Puchau et al., 2009). The absence of association discovered in the present research may be attributed to several factors, such as individual variations in antioxidant absorption and metabolism, as well as the complex interplay between antioxidants and the immune response to COVID‐19. Similarly, the DASH diet, which is known for its emphasis on fruits, vegetables, whole grains, lean proteins, and low‐fat dairy products (Appel et al., 1997), did not show a significant association with the clinical outcomes in COVID‐19 patients. The DASH diet has been linked with various advantages in health, such as lowering the risk of hypertension and cardiovascular diseases (Saneei, Salehi‐Abargouei, et al., 2014). However, according to the results of the present investigation, its impact on COVID‐19 outcomes remains inconclusive. It is possible that the DASH diet alone may not have a substantial effect on the inflammatory response and disease severity in COVID‐19, as the pathophysiology of the disease involves complex interactions between viral replication, immune response, and host factors (Schneider, et al., 2021). Alternatively, it may be worth exploring the potential association between a diet high in flavonoids and TMPRSS‐2, as previous studies have demonstrated the anti‐SARS‐CoV‐2 activity of various flavonoids, including hesperidin, amentoflavone, and narirutin. These flavonoids have shown the ability to bind to the active site of the TMPRSS‐2 protein, inhibiting its activity through hydrophobic and hydrogen bond interactions (Bellavite & Donzelli, 2020; Haggag et al., 2020; Varughese & Francis, 2022). However, in the present study, no significant relationship was discovered between dietary pattern scores and TMPRSS‐2. This lack of significance could be attributed to several factors, including the relatively small sample size, racial, and ethnic differences among participants, and the complex nature of the disease being investigated.

The present study demonstrated that adherence to a diet with higher dietary TAC or DASH diet score was associated with a decreased risk of COVID‐19. These findings are consistent with previous research indicating that high‐quality dietary patterns are linked with a lower incidence of COVID‐19 infection and shorter duration of hospitalization (Rahmati et al., 2023). In a prospective cohort study, Merino et al. demonstrated that dietary patterns characterized by healthy plant‐based foods were related with diminished risk and severity of COVID‐19 (Merino et al., 2021). Kim et al. found that individuals who reported adherence to plant‐based diets had a 73% reduced risk of moderate‐to‐severe COVID‐19 (Kim et al., 2021). Similarly, higher adherence to the Mediterranean diet has been linked with lessened the likelihood of severe COVID‐19, shorter hospitalization and recovery duration, and reduced inflammation markers (Zargarzadeh et al., 2022). However, contrary to prior research, our study did not find a significant relationship between dietary TAC, DASH diet score, and the severity of COVID‐19. This discrepancy in findings may be ascribed to differences in dietary patterns among adults compared to our study population, as well as our focus on overall dietary patterns rather than specific food groups and components. The hyperinflammatory syndrome triggered by COVID‐19 is a major contributor to disease severity, and numerous studies have investigated the interaction between nutrients and inflammation (Brighenti et al., 2005; Merad & Martin, 2020). The effect of COVID‐19 severity is a complex interaction involving multiple factors, including genetic predisposition, comorbidities, and overall health status (Yamamoto et al., 2021; Zsichla & Müller, 2023).

Numerous studies have also suggested that a diet rich in antioxidants can decrease the risk of viral infections, including COVID‐19 (Carr & Maggini, 2017; Hemilä, 2017). Furthermore, the DASH diet, which contains bioactive compounds, has been linked to a reduced risk of chronic and infectious diseases and mortality by improving immune system function (Phillips et al., 2019; Soltani et al., 2020). The observed association between dietary patterns and the risk of COVID‐19 may be explained by the influence of nutritional status on inflammation and the innate and adaptive immune response (de Araújo Morais et al., 2021; Salazar‐Robles et al., 2021). Previous studies supporting this hypothesis have indicated that an optimal intake of antioxidants can prevent oxidative damage, modulate inflammation, and consequently affect the risk of COVID‐19 (Puchau et al., 2009). A review study conducted by Iddir et al. concluded that diet and specific nutrients can enhance the immune system's defense against COVID‐19 by interacting with transcription factors involved in the inflammatory response, such as NF‐kB and Nrf‐2 (Iddir et al., 2020). Additionally, certain mechanisms, such as the interaction between vitamins and specific transcription factors, have been proposed. For example, vitamin A interacts with the retinoic acid receptor (RAR), which may play a role in immune function, while vitamin D interacts with both the vitamin D receptor and the cellular receptor ACE2, which is important for viral entry into host cells (Glaab & Ostaszewski, 2020).

Overall, the findings of this study suggest that adherence to the DASH diet and a higher TAC may dwindle the risk of COVID‐19 infection and lead to shorter hospitalization periods by enhancing the immune system and inflammatory response. However, it is important to conduct further research to validate these findings and gain a deeper understanding of the underlying mechanisms.

Several limitations warrant consideration when interpreting the findings of this study. First, the relatively small sample size of this study raises concerns about the generalizability of the results to broader populations. Additionally, the study's case–control design limits its ability to establish a definitive causal relationship between dietary patterns and COVID‐19 outcomes. Second, the reliance on participants' self‐reported dietary intake through FFQs introduces the possibility of recall bias, as individuals under mental stress due to COVID‐19 might misremember their food intake patterns. Moreover, the study's omission of other lifestyle factors like physical activity and smoking could mask potential confounding effects that could influence the results.

## 5 CONCLUSIONS

In conclusion, the present study adds to the growing body of evidence indicating that higher dietary TAC and DASH diet scores may be protective against infections and ameliorate the outcomes of the disease (as mentioned reducing hospital stay) and in this way, help improve people's quality of life by reducing morbidity, mortality, and health care costs. If future research confirms these results, healthy diets and a well nutritional status would be a useful strategy to prevent and manage COVID‐19.

## AUTHOR CONTRIBUTIONS

**Fatemeh Dibaseresht:** Conceptualization (equal); data curation (equal); formal analysis (equal); investigation (equal); supervision (equal); writing – original draft (equal). **Mohammad Alizadeh:** Data curation (equal); resources (equal); supervision (equal); writing – review and editing (equal). **Jalal Moludi:** Data curation (equal); formal analysis (equal); methodology (equal); supervision (equal); writing – review and editing (equal).

## CONFLICT OF INTEREST STATEMENT

The authors affirm that they have no conflict of interest.

## ETHICS STATEMENT

The protocol of study was approved by the Medical Ethics Committee at the Nutrition faculty of Tabriz University of Medical Sciences (IR.TBZMED.REC.1401.446).

## INFORMED CONSENT

Informed written consent was obtained from participants.

## Data Availability

The collected data supporting the findings of the present study are not publicly available due to ethical and privacy concerns but are available from the corresponding author upon request.
